# Recurrent Retroperitoneal Spindle Cell Sarcoma: A Challenging Case Report and Management Considerations

**DOI:** 10.7759/cureus.42658

**Published:** 2023-07-29

**Authors:** Raghu Halappa Nagaraj, Han Grezenko, Yogesh Raut, Samia Rauf R Butt, Chukwuyem Ekhator, Mohammad Ahsan Anwaar, Sophia B Bellegarde, S. M. Iram Shahzed, Archana Das, Abdullah Shehryar, Abdur Rehman

**Affiliations:** 1 Surgery, Avalon University School of Medicine, Willemstad, CUW; 2 Translational Neurosciences, Barrow Neurological Institute, Phoenix, USA; 3 Medicine, Narendra Kumar Prasadrao Salve Institute of Medical Sciences, Nagpur, IND; 4 General Practice, California Institute of Behavioral Neurosciences and Psychology, Fairfield, USA; 5 Neuro-Oncology, New York Institute of Technology, College of Osteopathic Medicine, Old Westbury, USA; 6 Internal Medicine, Combined Military Hospital Lahore Medical College and Institute of Dentistry, Lahore, PAK; 7 Pathology and Laboratory Medicine, American University of Antigua, St John's, ATG; 8 Internal Medicine, South Brooklyn Health, Brooklyn, USA; 9 Internal Medicine, North East Medical College Hospital, Sylhet, BGD; 10 Internal Medicine, Allama Iqbal Medical College, Lahore, PAK; 11 Surgery, Mayo Hospital, Lahore, PAK

**Keywords:** soft tissue tumors, oncology, atypical spindle cell/pleomorphic lipomatous tumor, recurrent spindle cell sarcoma, retroperitoneal spindle cell sarcoma

## Abstract

Soft-tissue sarcomas (STS) comprise a heterogeneous category of malignant tumors originating from mesenchymal tissue. Spindle cell sarcoma, characterized by its infrequent occurrence, poses diagnostic and therapeutic complexities owing to its rarity. We present a case of an 80-year-old male with a diagnosis of spindle cell sarcoma in the retroperitoneal space. The patient underwent midline exploratory laparotomy for tumor excision and was planned for postoperative chemotherapy. Unfortunately, the tumor recurred aggressively, leading to a fatal outcome. This case highlights the uncommon occurrence of retroperitoneal spindle cell sarcoma (RPSCS) and the importance of accurate diagnosis, appropriate surgical management, and adjuvant therapy.

## Introduction

Less than 1% of all adult malignancies are sarcomas, a rare type of malignant tumor originating from the mesenchymal tissue [1]. Based on the presumed tissue of origin and architectural pattern, the World Health Organization divides the category of soft-tissue neoplasms into >50 different histological categories. Undifferentiated soft-tissue sarcomas (STS) are a significant group [2]. Sarcomas typically manifest as a painless lump, and they seldom have distant metastases, particularly in the lungs [3].

One of the unusual types of undifferentiated STS is spindle cell sarcoma. Only a few cases have been documented in the medical literature due to their rarity [4]. Spindle cell tumors are uncommon, have a low incidence, and can develop in any portion of the human body, including the retroperitoneal space. Their histological characteristics include a mixture of fat cells and fibroblast-like spindle cells in a matrix of collagen and mucoid material. It may have a carcinomatous or neoplastic morphological appearance [5]. The objective of this case report is to present and discuss a difficult case of recurrent retroperitoneal spindle cell sarcoma (RPSCS), focusing on the diagnostic and treatment challenges related to this uncommon cancer.

## Case presentation

An 80-year-old male presented to the surgical outpatient department with the complaint of a mass in the left hypochondrium and intermittent constipation. The patient had first observed the mass two months ago. Mass had gradually increased in size. It was non-tender, extending from the left hypochondrium to the umbilical region. Umbilicus was inverted, and there were visible veins on the mass and the adjacent quadrants as well. Intermittent constipation was associated with the abdominal mass. His history was remarkable. He had been treated for a left-sided direct inguinal hernia five years ago. He had a left-sided orchidectomy due to an extra-testicular atypical lipomatous tumor three years ago.

The patient was vitally stable. A general physical examination revealed that the patient was blind in the left eye, secondary to a road traffic accident. No remarkable findings in the examination of other systems, namely nervous, respiratory, and cardiac, were found. A complete blood workup was done. No significant finding was present. Ultrasound of the abdomen and pelvis pointed toward a retroperitoneal mass. Based on this finding, a computed tomography (CT) scan of the chest, abdomen, and pelvis was performed. Along with it, levels of serum alpha-fetoprotein (AFP) and beta-human chorionic gonadotropin (hCG) were also measured. Both were within normal ranges (1.42 IU/mL and <2ng/ml). CT scan showed a large complex abdominal mass occupying most of the abdominal cavity, measuring 28.5 × 20.0 cm, having internal soft tissue density and fat density mass. The lesion was found pushing the left kidney superiorly, along with compression of descending colon. No remarkable finding in the chest or pelvic region was found. There was no lymphadenopathy. Furthermore, a core biopsy of the lesion was performed. Section revealed tissue cores with spindle cell neoplasm, composed of sheets and fascicles of moderately pleomorphic spindle cells with 15 mitoses per 10 high power fields. Immunohistochemical staining showed CD34 and S100 positive. Hence a diagnosis of spindle cell sarcoma was established. Based on the complete workup and established diagnosis of spindle cell sarcoma, midline exploratory laparotomy was performed. A mass measuring 21 × 20 cm was excised after separation from surrounding structures. Mass was 15 kg in weight, measured after the operation. The post-operative recovery of the patient was uneventful. Histopathology of the excised lesion confirmed the results of initial investigations (core biopsy). The patient was explained about the nature of his illness and the importance of regular follow-ups. The patient was directed to the oncology department for postoperative chemotherapy. However, the patient failed to comply with the surgeons’ advice even after repeated explanations and did not get an oncology consult.

**Figure 1:**
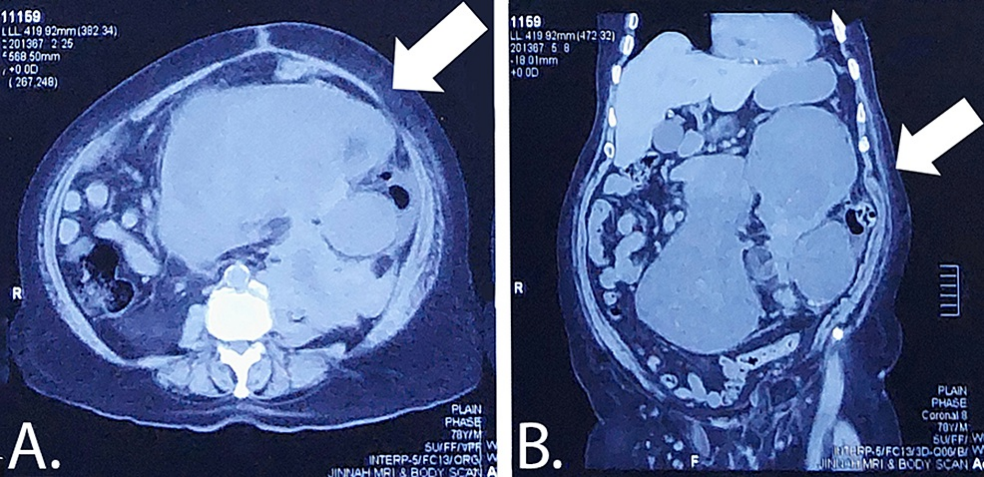
Abdomen CT scan. Part A (axial view) shows a hypodense mass in the abdomen and pelvis marked by the arrow. Mass can be seen arising from the left side, and bilobed morphology can also be appreciated. Part B (coronal view) shows a mass arising from the left hypochondrium and extending to the pelvic area, marked by the arrow.

The patient presented again four months later with similar complaints and additional complaints of urinary obstruction and lower back pain. Mass had recurred in the abdomen, and it was in the left hypochondrium. Mass had grown aggressively this time. Mass had started growing for one month (that is three months after the first laparotomy). CT scan showed 210 × 145 × 200 mm hypodense mass in the left hypochondrium and pelvis. It had a bilobed appearance with well-defined margins having internal necrosis and a solid component with no calcific or fatty component, causing compression over the left ureter leading to significant hydronephroureter. Details can be seen in Figure 1. Moreover, bone window images showed wedge-shaped compression collapse at L1 with significant disc osteophytic disease. Based on all the findings and the aggressive nature of the sarcoma, the multidisciplinary team decided to go with exploratory laparotomy and, after that, adjuvant chemotherapy. 

However, the patient’s vitals started crashing intraoperatively. A mass compromising of 10 kg was excised. The patient was shifted to intensive care unit (ICU) post-operatively. Unfortunately, the patient’s vitals did not recover, and the patient expired within 48 hours of the surgery.

## Discussion

To the best of our knowledge, limbs are where soft tissue tumors typically manifest. Abdominal soft tissue tumors are, therefore, uncommon, particularly retroperitoneal spindle cell tumors [6]. People of practically any age and sex can develop spindle cell sarcomas [7]. The median age at presentation was 57 years, according to two different investigations by Feng et al. and Smith et al. [2].

Due to their rarity, sarcomas sometimes undergo delayed or incorrect diagnoses, particularly in settings with limited facilities. A patient's history, imaging results, and biopsy are all part of the evaluation of a suspected soft-tissue sarcoma patient. In the case being presented, a CT scan and biopsy helped immensely in directing surgeons toward a definitive diagnosis. The key modality for evaluating soft-tissue masses in the extremities, trunk, head, and neck is magnetic resonance imaging (MRI) [2].

The recurrence of spindle cell sarcoma can vary depending on several variables, such as the location and stage of the tumor, the efficacy of treatment, and unique patient characteristics. Local recurrence following surgery was documented in two distinct investigations by Swamsura et al. and Diageler et al., with median follow-up times of 19 months and 15.7 months, respectively [8,9]. Surgery is the primary approach to treatment for RPSCS, with full tumor excision being the goal whenever possible [10]. The location and size of the tumor must be determined by preoperative imaging, such as CT or MRI. Based on tumor invasion, surgery may involve partial or total removal of nearby organs or structures. Postoperative radiation therapy can improve local control and lower the risk of recurrence by using methods like intensity-modulated radiation therapy (IMRT) or stereotactic body radiation therapy (SBRT) [11]. While there are few systemic treatment options, high-risk cases may benefit from adjuvant chemotherapy using drugs like doxorubicin, ifosfamide, or gemcitabine plus docetaxel. Regular post-treatment surveillance with imaging and clinical tests is required for monitoring and early intervention, and multidisciplinary teamwork between surgical oncologists, radiation oncologists, and medical oncologists is essential [12].

## Conclusions

STS must be viewed as a single entity to assist clinical research on these incredibly rare disorders. Due to the diverse natural history and biological behavior exhibited by STS, establishing management guidelines that adequately address the unique clinical presentations of individual patients is exceedingly challenging. With more than 50 distinct histologic subtypes, physicians must tailor guidelines to align with the specific clinical characteristics of each patient's disease. Nonetheless, certain fundamental principles form the basis for a multimodal approach to managing STS, which should be applied within the framework of a highly personalized assessment of both the patient and the tumor.

Publication of this case report on spindle cell sarcoma is essential to add to the limited body of knowledge on this uncommon and heterogeneous disease, providing valuable insights into its clinical presentation, management strategies, and potential therapeutic approaches, ultimately enhancing and improving the care for patients with spindle cell sarcoma.
